# Okra Mucilage as a Multifunctional Fat Replacer in Mozzarella Cheese: A Nutritious and Sustainable Solution for the Food Industry

**DOI:** 10.1002/fsn3.70858

**Published:** 2025-09-03

**Authors:** Rabbia Khan, Ali Ikram, Muhammad Tayyab Arshad, Feroza Naveed, Md. Sakhawot Hossain, Sammra Maqsood, Hatem A. Al‐Aoh, Kodjo Théodore Gnedeka

**Affiliations:** ^1^ University Institute of Food Science and Technology The University of Lahore Lahore Pakistan; ^2^ Functional Food and Nutrition Program, Faculty of Agro‐Industry Prince of Songkla University Songkhla Thailand; ^3^ Department of Nutrition and Food Technology Jashore University of Science and Technology Jashore Bangladesh; ^4^ National Institute of Food Science and Technology University of Agriculture Faisalabad Faislabad Pakistan; ^5^ Analytical Chemistry Research Laboratory, Department of Chemistry, Faculty of Science University of Tabuk Tabuk Saudi Arabia; ^6^ Togo Laboratory: Applied Agricultural Economics Research Team (ERE2A) University of Lomé Lome Togo

**Keywords:** dairy product, fat, milk, okra mucilage

## Abstract

Mozzarella is a white, soft, fermented cheese that is often recognized for its stretchability and typically contains approximately 40% total fat (dry basis), a considerable portion of which is saturated fat. Low‐fat mozzarella cheese (LFMC) has started to increase in popularity among health‐conscious consumers. Unfortunately, the inadequate meltability and rubbery texture of LFMC make it undesirable for many consumers. While excessive intake of certain types of fat, particularly saturated fat, has been associated with obesity and coronary heart disease, emerging research highlights the importance of fat quality and the role of healthy fats in the diet. This type of cheese is in high demand worldwide, particularly in pizza manufacturing. However, fat reduction often leads to undesirable textural and functional changes that could affect consumer acceptance. To avoid these issues, researchers are increasingly focusing on plant‐based fat replacers. Among these, okra mucilage (OM) has emerged as a potential candidate owing to its natural thickening properties and functional sugars. OM accounts for improved mouthfeel and texture, improving common problems in low‐fat cheeses, such as rubbery texture and poor meltability. It has been found that incorporating approximately 2.5% OM into LFMC can enhance stretchability and melting characteristics without sacrificing acceptable sensory qualities; however, consumer acceptance of taste and texture needs to be determined before the total replacement of dairy fat. Further research is needed to determine the optimum conditions for the use of OM to ensure batch‐to‐batch uniformity of cheese quality.

## Graphical abstract

Okra mucilage (OM) accounts for improved mouthfeel and texture, improving common problems in low‐fat cheeses, such as rubbery texture and poor meltability. It has been found that incorporating approximately 2.5% OM into low‐fat mozzarella cheese (LFMC) can enhance stretchability and melting characteristics without sacrificing acceptable sensory qualities. Further research is needed to determine the optimum conditions for the use of OM to ensure batch‐to‐batch uniformity of cheese quality.

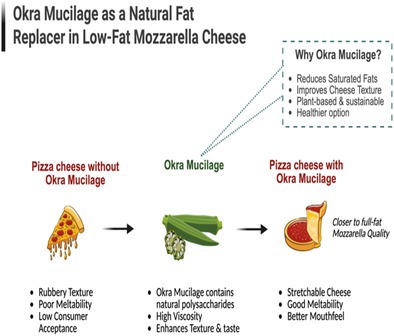

## 1 Introduction

Dairy products are important sources of nutrients that help maintain human nutritional and health requirements. They also contribute significantly to bone health and provide bioactive compounds that support immune functions (Pereira and Vicente 2017). Among the different fermented dairy products, cheese is a widely consumed and diverse fermented dairy product that is manufactured through casein coagulation. A typical global diet ranks second among suitable carriers of milk solids, including fats, proteins, and minerals, after fresh dairy (Bojovic and McGregor 2023).

The global production volume for cheese reached approximately 22 million tons in 2022, as the statistics indicated (Schädle et al. 2022). Currently, approximately 1500 cheese varieties have been documented globally. Notable examples include mozzarella, cheddar, ricotta, gorgonzola, cottage, gouda, edam, roquefort, parmesan, feta, Swiss, and Romano (Zhao et al. 2023).

Mozzarella cheese, an unripened cheese with a white glossy appearance and a soft to firm texture characterized by a smooth elastic consistency and containing 30%–40% milk fat, is widely used as a topping for pizzas (Gislon et al. 2023). Mozzarella, a cheese widely consumed globally, possesses a distinctive fibrous texture (Feng et al. 2023). The stringy appearance results from thermo‐mechanical treatment involving continuous kneading and stretching for a designated duration, leading to the elongation of protein fibers and aggregation of fat globules aligned with the stretching direction (Akhtar et al. 2022).

Italian legal standards stipulate that the commonly utilized mozzarella cheese (MC) must include at least 45% dairy fat and maintain a moisture content of 52%–55% on a dry basis (Hamad et al. 2017). Excessive use of saturated and trans fats leads to high triglyceride and low‐density lipoprotein (LDL) levels, which are related to obesity and the development of cardiovascular diseases (Froyen 2022). 17.9 million people die due to cardiovascular disease every year, as stated by the World Health Organization (WHO), accounting for 32% of total deaths. This is a significant health issue that necessitates proactive measures and adequate treatment to decrease the mortality rate associated with cardiovascular diseases (WHO 2021).

Consequently, reducing fat consumption has emerged as an urgent priority for national health authorities (WHO 2022). The MC varieties available in the market are categorized according to their moisture content. Fresh mozzarella cheese (FMC) has a moisture content of 52% or higher. Pizza makers favor low‐moisture mozzarella cheese (LMMC), with moisture contents varying from 42% to 45% (Alinovi et al. 2020). LMMC can be prepared from either whole milk or skimmed milk and further processed into shredded MC (Torrijos et al. 2022).

Reducing the fat content of cheese significantly alters its compositional balance, often resulting in undesirable properties such as poor meltability, rubbery texture, bland flavor, and off‐color appearance, compromising essential cheese functionalities such as stretchability, spreadability, and overall sensory appeal (Sun et al. 2021). In the pizza industry, higher functionalities are needed, and lowering the fat content of LFMC to below 10% compromises these essential functionalities. This type of mozzarella cheese tends to be more brittle, harder, less pliable, and more rubbery overall (Paximada et al. 2021). There is an opportunity to introduce new LFMC with greater marketability for healthier options (Giha et al. 2021).

Various strategies, such as modifying the production process and incorporating fat substitutes, may enhance the acceptance of low‐fat cheeses by improving essential functionalities, such as meltability, stretchability, texture, and flavor (Qi et al. 2025; Sattar et al. 2024; Hossain et al. 2025). Plant and animal‐based fat replacers (FRs) have been shown to replicate essential sensory, functional, and rheological properties in LFMC produced with low‐fat milk (Temkov and Mureșan 2021). They possess the capacity to mechanically retain water owing to their high hydrophilicity. Consequently, they are often suggested for formulating low‐fat cheeses. Furthermore, fat mimetics allow for reduced fat content in cheese, while maintaining mouthfeel creaminess (Bansal and Mishra 2020). In the food processing industry, several plant and microbial gums, such as alginate, guar gum, carrageenan, maltodextrins, pectin, mucilage, and xanthan gum, have been identified (Dantas et al. 2021).

Natural hydrocolloids have attracted interest over the past few years because of their functionality in food systems. Marine mucilage is derived primarily from microscopic algae known as phytoplankton, which exude mucilage as a result of different processes such as overgrowth, exudation, death, and decomposition. Conversely, mucilage derived from plants can be acquired under standard conditions, in contrast to marine mucilage, which is produced by organisms in response to damage and/or stress (Savun‐hekimoğlu and Gazioğlu 2021). Consequently, plant mucilage can be acquired with greater ease and is available in substantial quantities. In addition, it possesses green, sustainable, economical, renewable, and nontoxic advantages (Tosif et al. 2021).

Okra ( *Abelmoschus esculentus* ) is an economically important vegetable crop belonging to the *Malvaceae* family. Various parts of the plant, particularly pods, are widely used for both culinary and medicinal purposes. According to a case study on the composition of dried okra pods, it was discovered that each 100 g pod contains 9.63–13.33 g of moisture (w/w), 0.56–2.49 g of fats (w/w), and 10.25–26.16 g of crude protein (w/w) along with 36.66–50.97 g of carbohydrates (w/w) (Y. Liu et al. 2021). OM is composed of coiled polysaccharides such as galactose, galacturonic acid, and rhamnose. OM contains structural units defined by a number of disaccharide side chains, including (1–4)‐galacturonic acid and (1–2)‐rhamnose (Mohammadi et al. 2018).

Examining all accessible literature reveals a gap concerning the use of OM‐FR for LFMC formulation and a holistic approach for quality defect minimization and technological characteristic enhancement. Such gaps can be addressed by reviewing the literature concerning LFMC formulations using OM. This work presents an extensive review of the use of OM as a fat substitute in LFMC to achieve stable viscoelastic and functional properties.

## 2 Composition and Functional Properties of OM

OM contains polysaccharides, such as D‐galactose, L‐rhamnose, and galacturonic acid. It also contains proteins and minerals including calcium, magnesium, potassium, and iron, which contribute to its nutritional value. These components have a thick consistency along with gelling properties, making them useful in food and other industrial applications (Fatima et al. 2024).

### 2.1 Chemical Composition and Structural Characteristics

**FIGURE 1:**
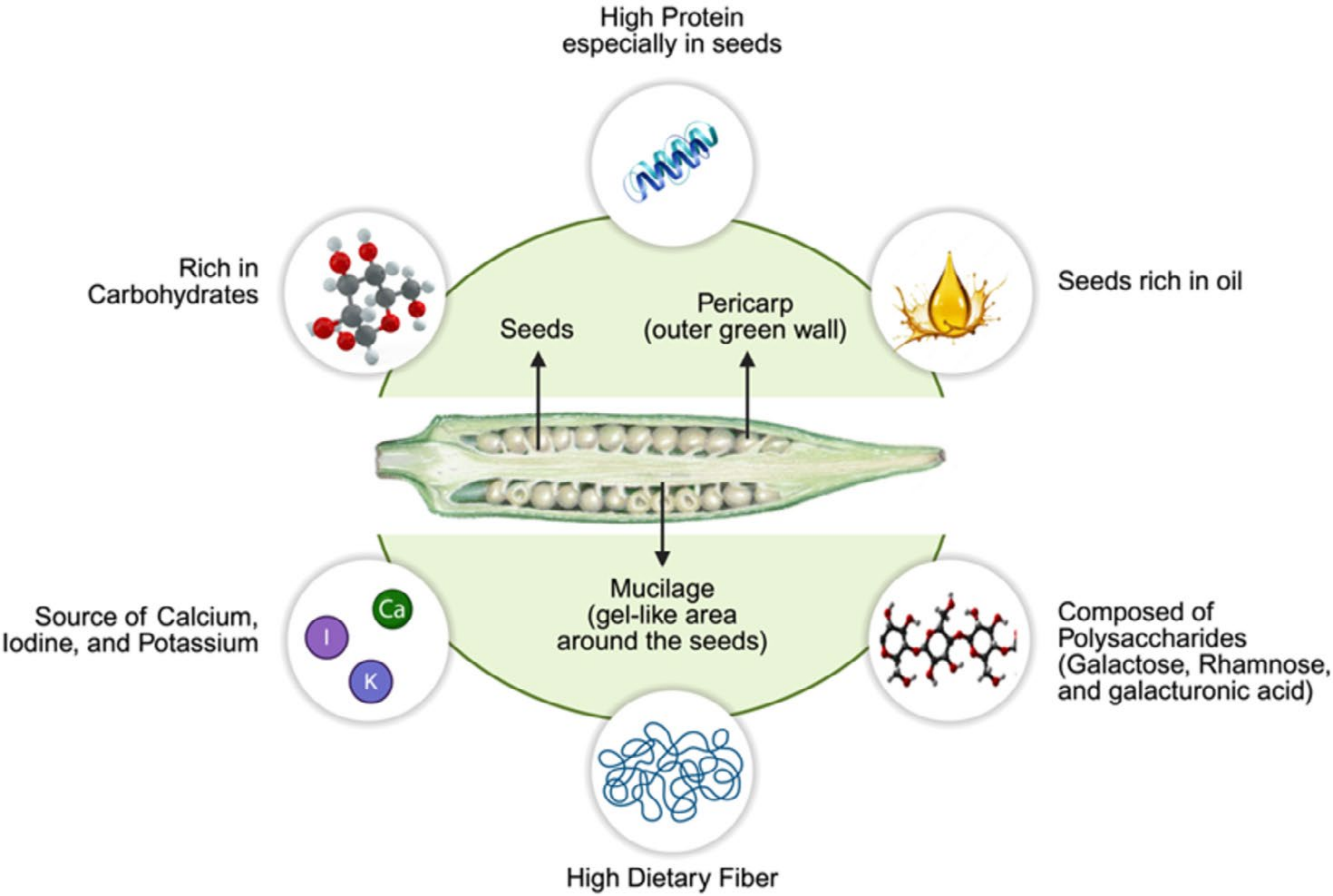
Structure and composition of okra pod.

Okra is a nutrient‐rich vegetable that contains a variety of essential vitamins and minerals, including calcium, potassium, B‐complex vitamins, amino acids, dietary fiber, and carbohydrates. It is also notably high in protein, particularly in seeds, which are recognized for their substantial protein and oil content (Basnet et al. 2023). Okra's composition includes flesh and seeds within a pod. The pericarp of pods is filled with mucilage polysaccharides that serve various biological purposes (Liu et al. 2018). The structural composition of polysaccharides in okra is primarily composed of galactose, rhamnose, and galacturonic acid, with small amounts of xylose and arabinose (Li et al. 2020). In addition to other sources, pectins in okra, primarily rhamnogalacturonan, have unique structural features that help it stand out (Figure 1) (Liu et al. 2018). Figure 1 shows the structure and composition of okra pod.

The primary structure of rhamnogalacturonan‐I contained both Rhamnogalacturonan I (RG‐I) and homogalacturonan, with remarkable differences in the RG‐I to HG ratio. The galacturonic acid content of pectin can be between 62.67% and 68.77%, which indicates that the amount of pectin present is considerable. Different methods of drying (freeze, sun, oven, and microwave) change the physicochemical features of fractions more than the other drying methods, with the viscosity and antioxidant properties of freeze‐dried pectins being the best (Xu et al. 2020).

Functional properties, such as emulsifying activity and water retention capacity, of Okra's polysaccharides are determined by their structural attributes, which differ with different extraction procedures and drying methods (Chen et al. 2021). The distinctive polysaccharide‐based mucilages in okra are abundant in their concentration, contributing to the thick and slimy consistency of water extracts, which is especially desirable for high‐quality okra soups. Mucilage is widely utilized in both the food and non‐food sectors as a mucilage thickener, fat replacer, and emulsion stabilizer (Afotey et al. 2023). Okra's high perishability renders it susceptible to phytochemical degradation due to both enzymatic and non‐enzymatic reactions, as well as to deterioration and spoilage resulting from the microbial presence (Afolabi et al. 2022).

However, the European Food Safety Authority proposed 25 g as the amount consumed each day for adults (Efsa 2017). Therefore, exceeding this amount is possible without surpassing the intake limit for 100 g of okra, achieving 32.6% of the average suggested values. Okra contains insoluble (4.73 g/100 g fresh weight) and soluble (3.43 g/100 g fresh weight) fiber. Both are associated with health benefits. Dietary insoluble fibers promote normal bowel movement and help prevent gastrointestinal diseases, especially diverticulosis of colonic segments. Soluble fibers undergo fermentation and modulation of lipid metabolism, resulting in lower cholesterol levels. Dietary fibers are responsible for the anti‐hyperlipidemic effects of okra on hyperlipidemia and associated metabolic disorders (Ciudad‐Mulero et al. 2019).

A related study found that the total polysaccharide content in okra pods was between 11.22 and 17.35 g per 100 g using the phenol‐sulfuric acid colorimetric method. The primary polysaccharide identified from the standard glucose curve was starch, whereas the phenyl–phenol method failed to detect pectic polysaccharides (Sami et al. 2019). Fresh okra pods have a limited shelf life; therefore, processing is necessary to prolong their usability and to address seasonal availability issues. Companies in developing countries utilize the sun and shade drying method for the off‐season use of okra pods (Nayak et al. 2015).

The presence of hydroxyl groups (–OH), including galacturonic acid and rhamnose, is notable in the polysaccharides found within OM. The presence of hydroxyl groups facilitated the formation of hydrogen bonds with water molecules, resulting in the formation of a gel network. Rhamnose and galacturonic acid, which are significant constituents of OM, possess carboxyl groups. The presence of these groups in the solution facilitates ionization, thereby enhancing the stability of the gel and enabling it to replicate the properties of fat through the formation of a viscous, stable matrix. The polysaccharides present in OM are interconnected through glycosidic bonds, resulting in a three‐dimensional network structure. This network captures water, leading to a gel‐like consistency (Fatima et al. 2024).

### 2.2 Rheological and Functional Properties

Okra polysaccharides exhibit shear‐thinning behavior and viscoelastic characteristics, whereas those extracted through water demonstrate pseudoplastic behavior influenced by their concentrations. OM solutions exhibit non‐Newtonian pseudoplastic behavior, characterized by flow behavior indices between 0.234 and 0.947, which signify shear‐thinning properties. The consistency coefficient fluctuated with concentration and temperature, exhibiting larger values at higher concentrations and lower values at higher temperatures. The activation energy for viscosity increases with concentration, indicating that elevated concentrations require greater energy for flow (Akcan and Glaue 2024).

Typically, galacturonic acid present in polysaccharides extracted from okra undergoes methyl esterification at C‐6 and/or O‐acetylation at O‐2 and/or O‐3 (Maalej et al. 2023). Consequently, okra polysaccharide demonstrates significant properties in preserving emulsion stability owing to its amphiphilic characteristics and viscosity. To date, the structural composition of polysaccharides has been largely elucidated, revealing that the primary chain consists of a repeating unit of →4‐ɑ‐GalAp‐(1 → 2)‐ɑ‐Rhap‐1→, while the side chain is characterized by β‐Galp‐(1 → 4). β‐Galp‐1 → Rhap residue O‐4 (Zhu and Obara 2022).

OM exhibits a more pronounced extensional viscosity than shear viscosity and is two to three orders of magnitude greater (Zhu and Mizunuma 2017). The decrease in viscosity with declining pH is attributed to the formation of larger polymer aggregates (Yuan et al. 2018a). The polydispersity index values that determine the range of viscosity differences confirm that the rheological properties of okra polysaccharides are of great functional versatility as fillers, thickeners, and gelling agents in food and pharmaceuticals (Ding et al. 2021). The decline in viscosity as the pH decreases is caused by the aggregation of polymers into fewer, larger entities (Yuan et al. 2018a). Moreover, 1% OM shows a viscosity close to 240 cP, at 247.34 cP for OM (Palei et al. 2016).

OM exhibits significant water‐holding capacity, constituting one of its primary functional properties (Gemede et al. 2018). The polysaccharides in mucilage, including galacturonic acid and rhamnose, contribute to this ability by forming a gel‐like network that traps and retains water (Kalkan and Maskan 2023). The water retention ability of OM can vary due to the extraction method, age of the okra, and other environmental factors (Noorlaila et al. 2015). It has a high water absorption capacity, which makes it useful in many food processes, such as thickeners and stabilizing agents in low‐fat formulations.

OM has important characteristics of thermal stability, which make it possible for the material to maintain its properties at high temperatures. This makes it suitable for many food processing applications (de Alvarenga Pinto Cotrim et al. 2016). To test the solubility of the mucilage, a solubility test in water and other organic solvents was performed. A 1% sample of the extracted mucilage was placed in polar and nonpolar solvents, including distilled water, acetone, chloroform, and ethanol. The results showed that mucilage was soluble in water and, as expected, insoluble in chloroform and acetone. The results of this study are in agreement with other research that showed that mucilage was more soluble in heated water than in cold water and was completely insoluble in all organic solvents (Farooq et al. 2013).

OM helps in emulsifying and thickening food formulations, such as tomato sauce, improving its acceptance and enhancing other sensory attributes (Table 1) (de Araújo et al. 2020).

**TABLE 1:** Role of plant‐based mucilages in fat replacement for healthier food products.

Food products	Fat replacer	Results	References
Mayonnaise	Canned white bean aquafaba	Low‐fat mayonnaise incorporated with aquafaba, the liquid from canned white beans, as a plant‐based emulsifier to facilitate the blending of ingredients. The product contained only 30% sunflower oil, contributing to its healthier profile. To maintain the thickness and enhance the texture of mayonnaise, supplementary ingredients such as thickeners and gelling agents incorporated. This method ensures that the mayonnaise remains smooth and achieves the appropriate consistency	Sachko et al. (2023)
Mozzarella cheese	Okra mucilage	Okra mucilage can effectively substitute for fat in preparing low‐fat mozzarella cheese Different levels of okra mucilage containing 0.25%–1.0% percent were analyzed. It was found that the addition of okra mucilage considerably decreased fat content in the cheese. For sensory evaluation, 0.25% concentration was found to be optimal. On the other hand, 1% concentration improved the texture and other important features of low‐fat mozzarella cheese. Okra mucilage was found to improve the lower‐fat mozzarella cheese's functional properties, while fat content reduction enhanced its nutritional value without compromising taste or texture	Akhtar et al. (2022)
Yoghurt	Tigernut milk and soybean milk	Tigernut milk and soybean milk to produce a plant‐based yoghurt. These ingredients serve as a fat substitute, enhancing the healthfulness of the yoghurt. The resulting yoghurt maintains high quality and retains its nutritional benefits, positioning it as a viable alternative to conventional cow milk yoghurt. This allows for the consumption of yoghurt that is low in fat while still providing essential nutrients	Adeyanju et al. (2025)
Yoghurt	Chia seed mucilage	The incorporation of 7.5% chia seed mucilage CSM into yoghurt mitigates the occurrence of watery separation during storage, in contrast to standard full‐fat yoghurts. This enriched yoghurt contains higher dietary fiber, rendering it more nutritious compared to both full‐fat and skimmed yoghurts. Tests indicated that the yoghurt containing 7.5% CSM exhibited increased thickness, firmness, and improved structural integrity, suggesting enhanced resilience under stress. Participants who sampled the yoghurt reported comparable levels of acidity, creaminess, and thickness to conventional yoghurts	Ribes et al. (2021)
Chicken meat model	Lemon albedo, mango peel powder and banana peel powder	Chiken gas emulsions can be produced using plant source fat replacer such as 1% lemon white albedo, 2% mango peel, and 2% banana peel powder. These ingredients replace 50% of the vegetable oil in the recipe This not only helps reduce fat but also improves the texture and consistency of chicken meat patties. The result is a healthier option that still has a good mouthfeel and quality	Chappalwar et al. (2022)

## 3 Extraction and Isolation of OM

Extraction of okra pod mucilaginous material was performed using the method described by Aziz et al. (2018). Okra pods were cut open along the longitudinal axis to remove seeds that were void of mucilaginous material. Slices were macerated in warm distilled water at 60°C for 12 h in a ratio of 1 part of the okra slice to 3 parts of water. This procedure shows good promise for mucilage extraction. After separation, mucilage was filtered through a clean muslin cloth. The mucilage obtained was stored in a dark container and refrigerated below 10°C. An aqueous medium is very useful for the extraction of OM because of the favorable yields and high efficiency, which is very beneficial in relation to this method of extraction. Okra polysaccharides have multiple hydroxyl groups (−OH), making the chemically modified surface of polysaccharides enormously diverse (Table 2) (Freitas et al. 2015).

**TABLE 2:** Comparison of chemical compositions of okra polysaccharides from different extraction methods.

Extraction methods	Monosaccharides	References
Hot water extraction	Galactose, rhamnose, arabinose, galacturonic acid	Liu et al. (2018)
Ultrasound	Galactose, rhamnose, arabinose, glucose, mannose, fructose, xylose	K. Wang et al. (2018)
Ultrasonic	Galactose, rhamnose, galacturonic acid	Nie et al. (2020)
Macerated	Galactose, rhamnose, galacturonic acid, glucose	Chen et al. (2016)

**FIGURE 2:**
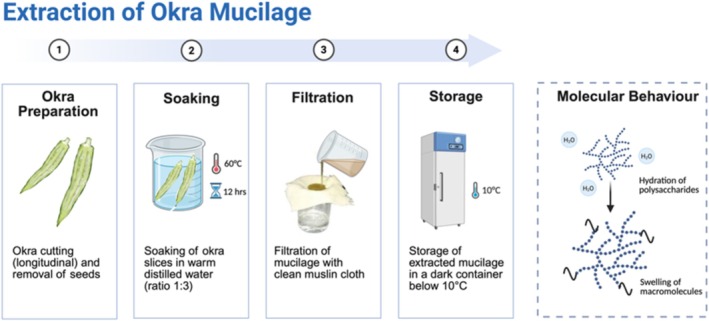
Extraction and isolation of OM.

The enhancement in the heat capacity led to an increase in the viscosity of the OM. Water extraction and mechanical‐assisted methods are increasingly being employed for polysaccharide extraction (Wang et al. 2018). Water extraction techniques include hydration of macromolecules in a solid framework. Following the interaction of macromolecules with aqueous solvents, these solid entities occupy space and undergo swelling. Subsequently, the expansion of solid materials facilitated the retrieval of macromolecules. Hydrated macromolecules interact in an aqueous phase, leading to reorganization that alters the rheological properties and transforms the phase into viscous hydrocolloids (Figure 2) (Ritzoulis 2017). Figure 2 shows the extraction and isolation of OM.

Cahyana and Kam (2016) assessed the impact of various factors, including time, temperature, water‐to‐okra fruit ratio, chemical composition, pH, extraction yield, and antioxidant and anti‐glucosidase activities. The various treatments applied during extraction did not have a significant impact on yield; nonetheless, the extract was obtained by soaking the fruit for 12 h at 4°C–5°C. The 1:6 ratio of fruit to water exhibited the highest antioxidant activity. The yield and biological activity of mucilage polysaccharides are influenced by factors such as time and temperature upon the extraction method employed.

### 3.1 Mechanisms of Fat Replacement in Cheese

While manufacturing LFMC, there are various options for substituting fat without altering the textures and functions of cheese. Recent studies have demonstrated that fat replacement can be effectively performed with plant sources, such as mucilages, oligosaccharides, and emulsifiers. This further enhances the texture and taste of cheese, making it more appealing to consumers with a low‐fat diet. Fat substitution aims to replace fat with non‐fat materials while maintaining full‐fat cheese characteristics, such as texture, flavor, and mouthfeel. The fat in cheese acts as a matrix filler, improving the creaminess, mouthfeel, and lubrication. A high level of fat removal can result in a product that is hard, rubbery, and bad tasting (Khanal and Bansal 2020).

Various techniques have been utilized to enhance the textural and flavor characteristics of LF cheese, with the use of fat substitutes being one of the most promising approaches. The application of three whey protein‐based fat replacers, Dairy‐Lo (purity index 35%), simpless (sincerity 53%), and LS1907 (purity index 90%), has been documented as an effective formulation of LFMC (Tahereh et al. 2017). Whey protein concentrates are widely utilized because of their high nutritional value and functional properties, including foaming, gelling, and emulsifying capabilities (Khalesi and FitzGerald 2021), enabling their application as fat replacers in various food systems (Li et al. 2021).

Fat replacers enhance functional attributes, particularly texture smoothness, fatty mouthfeel, and viscosity, while also influencing the oral and flow properties of low‐fat food products. They provide precise fat‐like functional characteristics for LF food products (Tahereh et al. 2017). In food processing industries, plant and microbial gums, such as alginate, guar gum, carrageenan, maltodextrins, pectin, mucilage, and xanthan gum, are extensively utilized as polysaccharide stabilizers to limit water mobility and enhance fatty mouthfeel (Dantas et al. 2021).

The use of some FRs made from plant and animal sources has been proven to mimic important sensory, functional, and rheological properties of LFMC made with skim milk (D. Zhang 2022). Different FRs were studied for their effects on the functional, rheological, flow, melting, and lubricating properties. This involves precise formulation and testing to ensure that the final outcomes meet the market's expectations and requirements.

### 3.2 Impact on Cheese Properties

#### 3.2.1 Textural and Functional Properties

Incorporating fat substitutes into mozzarella cheese alters its texture, balancing health demands and high quality. *Aloe vera* mucilage (AVM), whey proteins, and basil seed mucilage have been studied to evaluate their potential for fat reduction in mozzarella cheese. These substitutes serve the function of fat by improving stretchability, meltability, and other important structural attributes that are absent in low‐fat foods.

Studies have indicated that AVM can improve the textural properties of LFMC (Öncü Glaue et al. 2023). AVM at a concentration of 2.5% was shown to greatly enhance stretchability and meltability, while reducing hardness, thus improving consumer acceptability. Research has also suggested that AVM could effectively lower fat content while sustaining favorable organoleptic attributes that aid in consumer acceptance (Akhtar, Ansar, et al. 2024; Akhtar, Araki, et al. 2024).

Inulin and maltitol significantly increased elasticity and decreased hardness. Maltitol notably enhanced stretchability and lowered the glass transition temperature, thereby positively influencing the texture of cheese. The incorporation of fat substitutes, lecithin and maltitol, resulted in reduced hardness and increased free fat, thus improving texture (H. Li et al. 2019).

Whey protein concentrates, including CH‐4560 and YO‐8075, have been used to mitigate textural deficiencies in LFMC. These proteins enhance meltability and stretch performance, which are generally inadequate for low‐fat variants. Pre‐acidification in conjunction with whey proteins can partially ameliorate the hardness and low meltability of LFMC; however, if not optimized, it may adversely affect stickiness and stretch performance (Zhang, Ling, et al. 2024; Zhang, Xiong, et al. 2024).

Basil seed mucilage (BSM) at a concentration of 2.5% enhances the stretchability and meltability of LFMC, comparable to those of AVM. Microstructure analysis demonstrated that BSM can infiltrate the three‐dimensional network of cheese, enhancing its textural properties while maintaining a relatively stable hardness level (Akhtar, Ansar, et al. 2024; Akhtar, Araki, et al. 2024). Fat replacers mitigate health issues while preserving the sensory attributes expected in mozzarella cheese, including stretch and meltability (Akhtar et al. 2023).

Another study presented textural analysis of LF mozzarella cheese samples incorporating OM. The hardness (N) of the LF mozzarella cheese samples was assessed on various days (0, 3, 5, 7, and 9) of the ripening process at 4°C. Shear and puncture forces were employed to assess the hardness, which increased with the duration of storage. The hardness of all the LFMC treatments consistently changed during storage. Textural analysis indicated that the hardness was significantly greater (*p* = 0.043) on the 9th day of storage. The mozzarella cheese produced from skimmed milk is far harder than cheese produced using sodium alginate as a fat replacer (Chatli et al. 2019).

The fat content of milk directly influences the meltability of mozzarella cheese. An increase in fat content improves the meltability and softness of mozzarella cheese. LF milk typically results in mozzarella cheese of inferior quality, characterized by undesirable color and rubbery texture (Ah and Tagalpallewar 2017). Akhtar et al. (2022) demonstrated that the incorporation of a fat substitute (OM) significantly influences the meltability of LFMC samples. The meltability of all mozzarella cheese treatments ranged from 42 to 55 mm, measured by the diameter of the cheese spread after heating at 90°C for 5 min. The meltability of LFMC increased with increasing concentrations of fat substitutes. On the other hand, the stretchability ranged from 33.83 to 38.57 mm.

Full‐fat mozzarella cheese (FFMC) exhibited a meltability of 55 mm, while okra mucilage mozzarella cheese (OM‐MC) with a concentration of 1.0% showed a meltability of 49.10 mm. The meltability of LF mozzarella cheese increases with higher concentrations of fat substitutes. Stretability is a critical attribute when considering the application of mozzarella cheese in different dishes. The stretching quality of cheese is a crucial factor in determining the end use of mozzarella cheese in various food products. The United States Department of Agriculture (USDA) states that mozzarella cheese, when applied to pizza and baked at 425°F for 12 min in a conventional oven, should stretch to a minimum of 3 in. without breaking. The stretchability results for all the prepared mozzarella cheese samples stored on the third day were obtained under elevated temperature conditions (Zhu et al. 2015).

The mozzarella cheese samples exhibited stretchabilities ranging from 33.83 to 38.57 mm.

FFMC exhibited a meltability of 55 mm, whereas OM‐MC, with a concentration of 1.0%, showed a meltability of 49.10 mm. The meltability of LF mozzarella cheese increases with higher concentrations of fat substitutes (Akhtar et al. 2023).

#### 3.2.2 Sensory Attributes

LFMC is becoming more popular owing to health concerns related to high‐fat diets. However, when fat is reduced, it can affect texture, meltability, and flavor. Many studies have investigated the use of fat substitutes to improve the sensory qualities of low‐fat products. These studies show the effectiveness of different fat replacers in making low‐fat mozzarella taste and feel better (Öncü Glaue et al. 2023).

*Aloe vera* mucilage serves as an effective fat substitute in LFMC, demonstrating notable enhancements in stretchability and meltability, especially at a concentration of 2.5% v/v. This concentration resulted in the lowest hardness, which improved the organoleptic properties of the cheese (Akhtar et al. 2022).

The inclusion of soy and pea protein hydrolysates (PPHS) in LFMC enhances baking performance, stretchability, and sensory attributes. The incorporation of PPHS reduces texture parameters, including hardness and stickiness, thereby enhancing the sensory experience (Gao et al. 2022).

BSM at a concentration of 2.5% v/v enhanced the stretchability and meltability of LFMC. Analysis of the microstructure indicates that BSM can permeate the three‐dimensional network of cheese, thereby improving its sensory characteristics. Higher concentrations may enhance textural hardness over time (Akhtar, Ansar, et al. 2024; Akhtar, Araki, et al. 2024).

The addition of Guar Gum significantly improved the sensory properties of low‐fat mozzarella. Sensory evaluation performed post‐processing and during storage revealed improved texture and flavor attributes relative to the negative control (C–treatment). Guar Gum treatment enhances moisture content and improves overall sensory acceptance, thereby increasing the appeal of cheese while decreasing its energy value.

The sensory properties of LFMC, particularly with a reduction in fat from approximately 22% to 11% and salt from approximately 1.7% to 1.0%, demonstrate increased hardness and chewiness in unheated cheese. Reduced‐fat, reduced‐salt cheese demonstrated decreased flowability and increased work to extend (EW) upon heating. Reducing the calcium content enhances moisture levels and decreases hardness; however, the sensory attributes of LFMC are firmer and more cohesive than those of full‐fat, full‐salt cheese (Henneberry 2019).

The application of fat replacers and homogenization processes significantly changed the sensory attributes of LFMC. Assessors indicated reduced color scores for low‐fat cheese compared to full‐fat cheese. Unhomogenized samples exhibited greater stretchability; however, homogenization typically improved the other rheological properties. The addition of whey protein fat replacers to the product improved the moisture content, which in turn improved all sensory attributes. The lack of adequate texture and flavor renders reduced‐fat cheese poorly accepted by consumers (Tahereh et al. 2017).

The sensory characteristics of LFMC incorporating fat substitutes were more favorable than those of sodium alginate‐treated cheese (LFMC‐SA), which scored the highest in flavor and general acceptability over carboxymethyl cellulose‐treated cheese (LFMC‐CMC), which scored the lowest. All FFMC surpassed the LFMC variants in all sensory attributes (appearance, flavor, texture, and juiciness); however, LFMC‐SA showed the most promise with regard to sensory attributes. Juiciness and texture were significantly superior in LFMC‐SA to LFMC‐CMC, suggesting a better overall profile (Chatli et al. 2019).

The sensory characteristics of LFMC are notably influenced by the reduction of fat, resulting in diminished meltability, insufficient flavor, and unfavorable color. In terms of sensory evaluation of color, the scores fell within the range of 5–7.5, with flavor evaluations scoring between 5 and 7 on a nine‐point hedonic scale. Adding hydrocolloids, especially guar gum and xanthan gum at concentrations of 0.15%, improved the functionality and yield of low‐fat cheese and therefore brought the sensory properties close to those of full‐fat cheese (Sattar et al. 2015).

Organoleptic analysis revealed that 0.25% of patients received a score higher than 0.5% and 1%. This study demonstrated optimal outcomes at lower concentrations in organoleptic analysis, achieving comparable textural and functional properties at a concentration of 1% relative to commercially available LF mozzarella cheese. It can be concluded that the addition of specific flavors and colors, classified as food conditioners, along with 1% OM as a fat substitute, enhances organoleptic properties (Akhtar et al. 2022).

The organoleptic color scores for LF cheese samples ranged from 5 to 7.6, in comparison to T0. Mucilage from Okra demonstrated sensory attributes comparable to those of actual cheese, particularly in samples T2 and T3. The color of cheese is influenced by the type of milk used, fat content, packaging, and storage conditions (Bekele et al. 2019). Sattar et al. (2015) noted that xanthan and guar gum did not contribute to the flavor characteristics of LFMC compared with standard cheese.

## 4 Nutritional Benefits of Using OM

Incorporating OM into food products as a fat substitute enhances the nutritional value while maintaining flavor and consistency. Owing to its possible health advantages, it can be consumed untreated, regarding it as a functional food (Dantas et al. 2021). Recent studies on okra polysaccharides have focused mainly on their biological functions, including antioxidant, anti‐tumor, and anticancer activities (Table 3) (Musthoza et al. 2023).

**TABLE 3:** Uses of okra polysaccharides in biological studies.

Sr.	Okra Polysaccharide activity	References
1.	Showed a strong ability to fight against harmful free radicals.	Zhang et al. (2020)
2.	Exhibited antioxidant activity and in vitro tests, inhibited the actions of α‐amylase and α‐glucosidase	Wang et al. (2020)
3.	Caused different changes in the shape of gelatin when mixed in a specific process called complex coacervation	J. Li et al. (2020)
4.	Caused a decrease in blood sugar levels	Liu et al. (2018)
5.	Helps reduce tiredness or fatigue	Gao et al. (2018)
6.	Improves the immune system's ability to fight off infections. This includes boosting the activity of immune cells that consume harmful substances, increasing the size of the spleen, helping immune cells grow and multiply, and regulating immune responses by producing special signaling molecules called cytokines	Wahyuningsih et al. (2018)
7.	Created thin films of zinc oxide that included okra mucilage. These films were very effective at killing bacteria, showing stronger antibacterial effects against *Staphylococcus aureus* compared to *Escherichia coli*	Mohammadi et al. (2018)
8.	Showed strong antioxidant effects when tested in a lab	K. Wang et al. (2018)
9.	Help treat metabolic diseases by blocking certain signals in the body, specifically LXR and PPAR pathways	Fan et al. (2014)
10.	Significantly slowed down the growth of cells but increased the number of healthy cells surviving	(Deters et al. 2005)

Carbohydrates that are nutritionally enhanced in the variety of okra are called mucilage. Mucilage is a source of dietary fiber that enhances gut health and facilitates digestion. Moreover, bioactive compounds that may possess antioxidant characteristics and contribute to general well‐being are likely present in mucilage. In particular, okra's edible constituents, especially mucilage, are inexpensive and easily accessible, making them important for a healthy diet, especially in the tropical and sub‐tropical regions.

**FIGURE 3:**
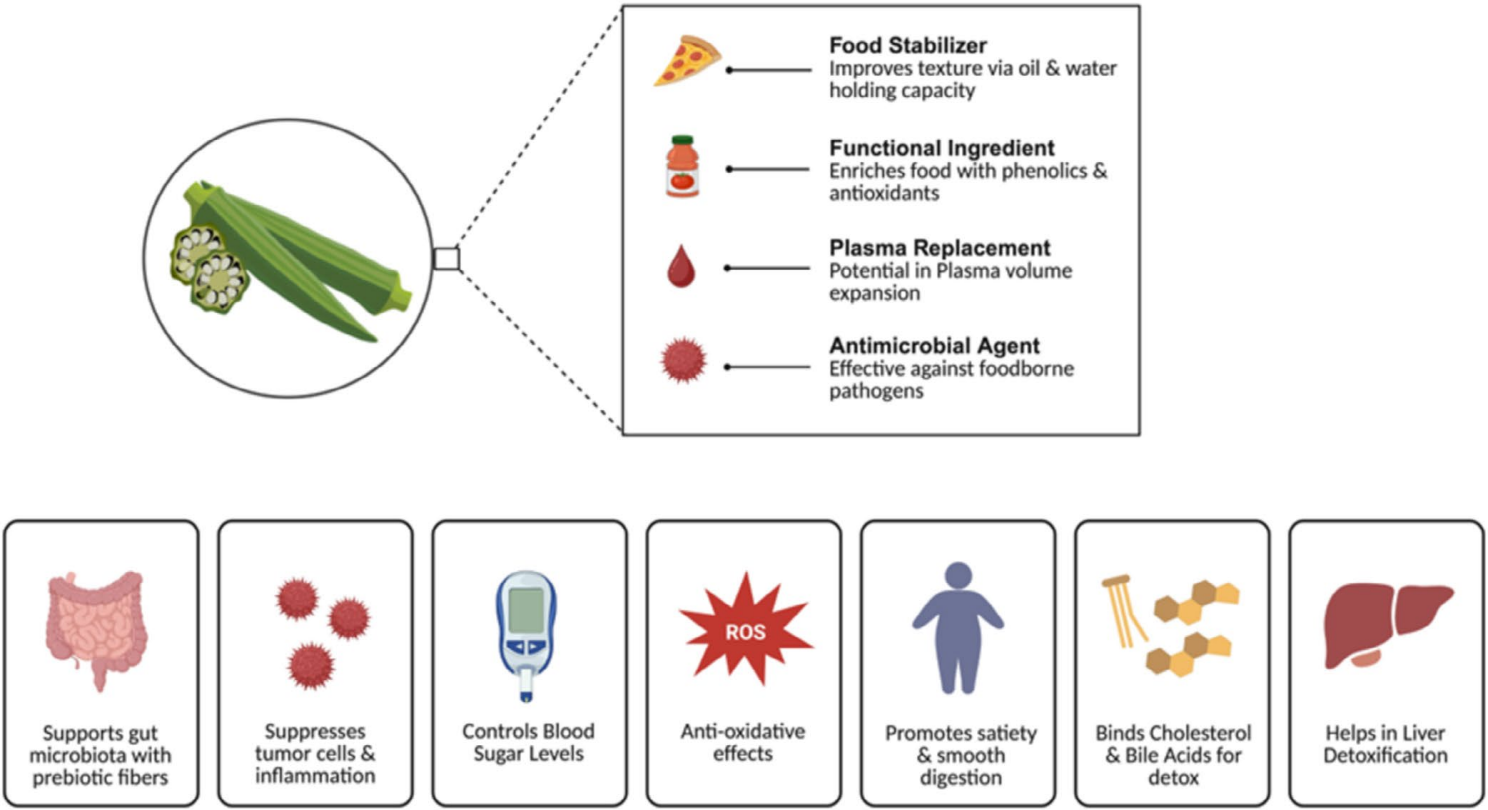
Industrial applications and health benefits of OM.

These polysaccharides from mucilage have important biological activities, such as immunomodulation and anti‐inflammation. There is strong evidence of the substantial functional health properties of OM from in vitro and in vivo studies, such as its capacity to bind cholesterol and bile acids, enabling the body to eliminate toxins from the liver along with other antitumor, antioxidant, antimicrobial, hypoglycemic, and antiulcerogenic (Figure 3) (Wahyuningsih et al. 2018). Figure 3 shows the industrial applications and health benefits of OM.

OM is known to contain naturally occurring polysaccharides and proteins in high concentrations. The abundance of vitamins and minerals makes their nutritional profile surprisingly impressive. Food products that contain monosaccharides such as D‐Galactose, L‐Rhamnose, and Galacturonic Acid show a beneficial nutritional profile. OM promotes the health benefits of better insulin resistance, anti‐cancer, and anti‐hypertensive effects and is known to contain bioactive substances linked with antimicrobial properties. The addition of moringa leaves into food matrices can serve as a beneficial component for health‐conscious consumers, as it enhances food properties (Fatima et al. 2024).

Potential biological activities, especially controlling biochemical parameters of type two diabetes, have been widely researched in the functional properties of OM (Liu et al. 2018). It contains three major components: beta‐glucan, resistant starch, and prebiotic fiber, which promote its activity by supporting the gut microbiota (Wang et al. 2025). The functional oil‐ and water‐holding capacity of mucilage enhances its application in the food industry. Its amino acid profile includes asparagine, proline, glutamine, and threonine, which provide adequate nutrition (Gajadhar 2023).

The macromolecule is being studied for its therapeutic efficacy in chronic disease management. These include liver detoxification, antibacterial interventions, plasma replacement, and blood volume expansion. The bioactive components of OM support digestive health and nutrient absorption, making it a useful candidate for a balanced diet aimed at sustainable health applications (Elkhalifa et al. 2021).

Lyophilized OM has a higher concentration of protein, sugar, and soluble solids than both fresh and wet mucilage. The observed tomato sauces that had OM had a high phenolic content along with high antioxidant capacity, as seen in the lyophilized mucilage variant, which measured 27.37 eq. trolox mg/100 g. This shows that the addition of OM enhances the nutritional value of tomato sauce while also providing beneficial bioactive matter (de Araújo et al. 2020).

Okra is rich in vitamins (including vitamins A, C, K, and B‐complex vitamins, such as B1, B2, and B6), minerals, and dietary fiber, making it a valuable addition to nutrient‐dense food sources. The mucilage contained in okra is helpful for weight loss because it aids in digestion, controls blood sugar, and induces feelings of fullness (Lesa et al. 2023). Bone health, along with heart and blood vessel health, is supported by the high antioxidant, calcium, and potassium content in okra, which together help fight oxidative stress and inflammation (J. Singh and Nigam 2023).

The nutritional value of OM is high because of its soluble fibers, which reduce serum cholesterol levels and stabilize body glucose levels by regulating sugar absorption in the intestine. It detoxifies the body by binding and removing bile acids and cholesterol. It also serves as a source of vital amino acids and polysaccharides, thereby improving general health. It is also used in cooking as a viscous ingredient to make different forms of foods smoother, while possibly preventing digestive issues and lowering the risks of obesity and other chronic diseases (Gemede et al. 2015).

Its non‐toxicity, low cost, and compatibility make it suitable for multiple applications (Dantas et al. 2021). Mucilage also has high levels of iodine, which help treat goiter disease (Singh et al. 2017).

The findings of the study by (Nampuak and Tongkhao 2020) revealed a content of total phenolic compounds amounting to 11.33 mg of gallic acid per 100 mL and total flavonoids of 5.06 mg of catechin per 100 mL. Furthermore, OMP has potent antibacterial activity against food‐related pathogens, making it a valuable functional ingredient for the promotion of health in foods, particularly drinks.

The increased viscous properties of OM enhanced the inhibition of glucose trapping in somatic cells, as well as intestinal sugar absorption. This was demonstrated by the comparative anti‐hyperglycemic activities of amadumbe ( *Colocasia esculenta* ) and OM, where Chukwuma et al. (2018) highlighted the stronger anti‐hyperglycemic action of okra relative to amadumbe as a result of greater mucilage viscosity.

Reactive oxygen species can inflict irreversible cellular damage, but natural polysaccharides found in plant mucilages can be used as good antioxidants because they effectively reduce the damage. They not only eliminate free radicals, but also strengthen superoxide dismutase (SOD), thereby enhancing the antioxidant mechanism (Slima et al. 2018).

OM is known to contain carbohydrates, specifically the pectic polysaccharide fraction WOP‐2, which possesses a rhamnogalacturonan I backbone with Type II arabinogalactan side chains partially substituted at the O‐4 of rhamnopyranosyl. This specific structure might be important in reducing lipoperoxidation reactions that cause the destruction of beta cells (Zhang et al. 2018).

### 4.1 Other Properties of OM

OM demonstrates strong antioxidant properties and anti‐fatigue effects, implying that it can overcome body‐harming substances and alleviate fatigue. It is also beneficial for encapsulating probiotic bacteria, which are beneficial for digestion (Table 4).

**TABLE 4:** Antioxidant and anti‐fatigue attributes of okra mucilage.

Functional properties	Active compounds	Effects	Study	References
Antioxidant property	Polysaccharide	The soluble part of okra mucilage was tested for its ability to neutralize DPPH radicals, which are a type of free radical. The IC50 value of 785.5 μg/mL means that at this concentration, the okra mucilage can reduce the amount of DPPH radicals by half. This shows that okra mucilage is quite effective at scavenging or removing these harmful radicals, which is a good sign for its potential health benefits	In vitro	Khan et al. (2023)
	Polyphenol and flavonoid content	The overall content of phenolic compounds found in the mucilage of okra lies in the range of 4.66 to 49.93 mg of gallic acid equivalent per gram (mg GAE/g) and the content of flavonoids is in the range of 5.06 to 18.72 mg of catechin equivalent per gram (mg CE/g) When the levels of phenolics and flavonoids are higher, the ability of okra mucilage to fight off harmful free radicals also increases The effective concentrations (EC50) for its ability to scavenge DPPH radicals, which indicates how much is needed to be effective, range from 3.15 to 6.60 mg/mL	In vitro	Gemede et al. (2018)
Anti‐fatigue activity	Polysaccharide	Okra polysaccharides have been found to help mice swim longer, which suggests they may boost endurance. Additionally, they reduce the blood levels of urea nitrogen and lactic acid which are markers of lower stress and fatigue in the body. Additionally, these polysaccharides increase the amount of glycogen stored in the liver and muscles. Glycogen is a form of energy that the body uses during physical activity	In vivo	Y. Li et al. (2020)
	Polyphenols and flavonoids	Okra mucilage contains unique substances called flavonoids and polyphenols. These substances are advantageous because they lessen fatigue and guard against metabolic issues. Rat studies have demonstrated that these qualities may improve general health and vitality	In vivo	Fouda and Mohamed (2024)

#### 4.1.1 Antioxidant and Anti‐Inflammatory Properties

The outstanding anti‐inflammatory and antioxidant characteristics of okra mucilage make it useful in food products, including cheese. The mucilage from the pods of okra exhibits a great degree of antioxidant activity; the overall health benefits increase with the total content of phenolics and flavonoids (Gemede et al. 2018). The influence of various thermal processing techniques on bioactive constituents and their associated antioxidant activities has been widely studied, as it greatly depends on the temperature and time of heating. Many authors have noted a decline in antioxidant phenolic compounds in a number of cooked vegetables such as broccoli, carrot, tomato, potato, spinach, artichoke, zucchini, and beans (Nayak et al. 2015).

The antioxidant protective action of the polysaccharides of plants is extraordinarily helpful in inhibiting oxidative damage to the cellular processes of organisms by reactive oxygen species. In addition, these polysaccharides have the ability to enhance the activity of superoxide dismutase (SOD) in addition to free radical scavenging, thereby strengthening antioxidant defense (Slima et al. 2018).

It is best known for its active phenolic compounds that have antioxidant effects. Research suggests that the mucilage displays significant radical scavenging with effective IC_50_ values that are indicative of free radical scavenging activity (Chukwuma et al. 2018). The pectic polysaccharide WOP‐2 contains a carbohydrate portion that includes the backbone of rhamnogalacturonan and partially substituted type II arabinogalactan side chains at the O‐4 position of rhamnopyranosyl. This composition is known to possess antioxidant activity, which may attenuate lipid peroxidation reactions that damage beta cells (Zhang et al. 2018).

Moreover, it has been proposed that certain pectinic polysaccharides suppress the pro‐inflammatory cytokines TNF‐α and IL‐1β, thus providing a degree of anti‐inflammatory action on macrophage cells (M. Li et al. 2022). Polysaccharides and phenolic compounds, with the latter serving as the main bioactive constituent, predominantly make up okra. There is a wide array of phenolic compounds, including catechin, isoquercitrin, protocatechuic acid, quercetin, quercetin‐3‐O‐gentiobioside, and rutin (Wu et al. 2020). OM is rich in antioxidant and anti‐fatigue effects, with studies pointing towards possible benefits in reducing oxidative stress and improving stamina. However, the optimal dietary intake required to confer these health benefits is unknown and likely depends on the food matrix and eating habits.

#### 4.1.2 Potential for Probiotic Encapsulation and Gut Health Improvement

OM enhances the survival of probiotics during their transit within the digestive system and helps bacteria vital for health. This indicates that OM is potentially useful for the development of functional foods, which are foods intended to be nutritious beyond their basic value.

The possible benefits of mucilage okra for microencapsulating *Lactobacillus plantarum* for the stability and viability of probiotics are great. It might be possible for any gastrointestinal problem to be resolved by the effective delivery of useful bacteria that help to balance the microbiome and thus improve gut health (Pourakbar et al. 2023). Animals treated with okra polysaccharide microcapsules had greater levels of Lactobacillus, which is a marker for better gut health (Hsiao et al. 2024).

Mucilage from Okra can encapsulate *Lactiplantibacillus plantarum*, which improves the gut microbiota of Alzheimer's mice by increasing the number of *Lactobacillaceae* and *Lactobacillus*. This provides encouragement for encapsulated probiotics and gut health improvement (Hsiao et al. 2024).

The merging of two polymers may improve the structural and physicochemical changes of the matrix and, in turn, alter the size attributes, encapsulation effectiveness, release rate of the drug, and the biopharmaceutical properties of drugs (de Medeiros et al. 2013). Controlled release of oxcarbazepine was achieved in microspheres made of OM and alginate (Ghumman et al. 2018). The pharmacokinetic parameters of the formulations were markedly different from those of the individual drugs.

Controlled release tablets of lamivudine were formulated with different quantities of OM excipient (Palei et al. 2016). The results showed that the concentration of mucilage in the in vitro release decreased, illustrating its capacity to modulate drug delivery from the matrix.

## 5 Sustainability and Economic Aspects of OM Utilization

Okra is a sustainable crop because it does not require much water and does not rely heavily on chemical pesticides. This vegetable grows well in dry areas and can be cultivated with little environmental impact. Because of these traits, okra is a practical option for farming in a way that is better for the planet.

### 5.1 Environmental Benefits of OM

In addition to its functional properties in food applications, OM offers environmental advantages due to the sustainable nature of okra cultivation. This section outlines the benefits to support the potential of okra‐based ingredients in eco‐friendly food production. The application of indole acetic acid (IAA)‐producing rhizobacteria enhances drought resilience in okra, revealing notable enhancements in both shoot and root growth, germination rates, and root colonization in water‐deficient conditions (Mahmood et al. 2025). Mohapatra et al. (2024) reported that integrated (IM), biointensive, and traditional chemical (CM) pest management strategies for okra farming demonstrate that IM markedly diminishes insect damage and maximizes crop yield, but CM adversely affects natural enemy populations.

**FIGURE 4:**
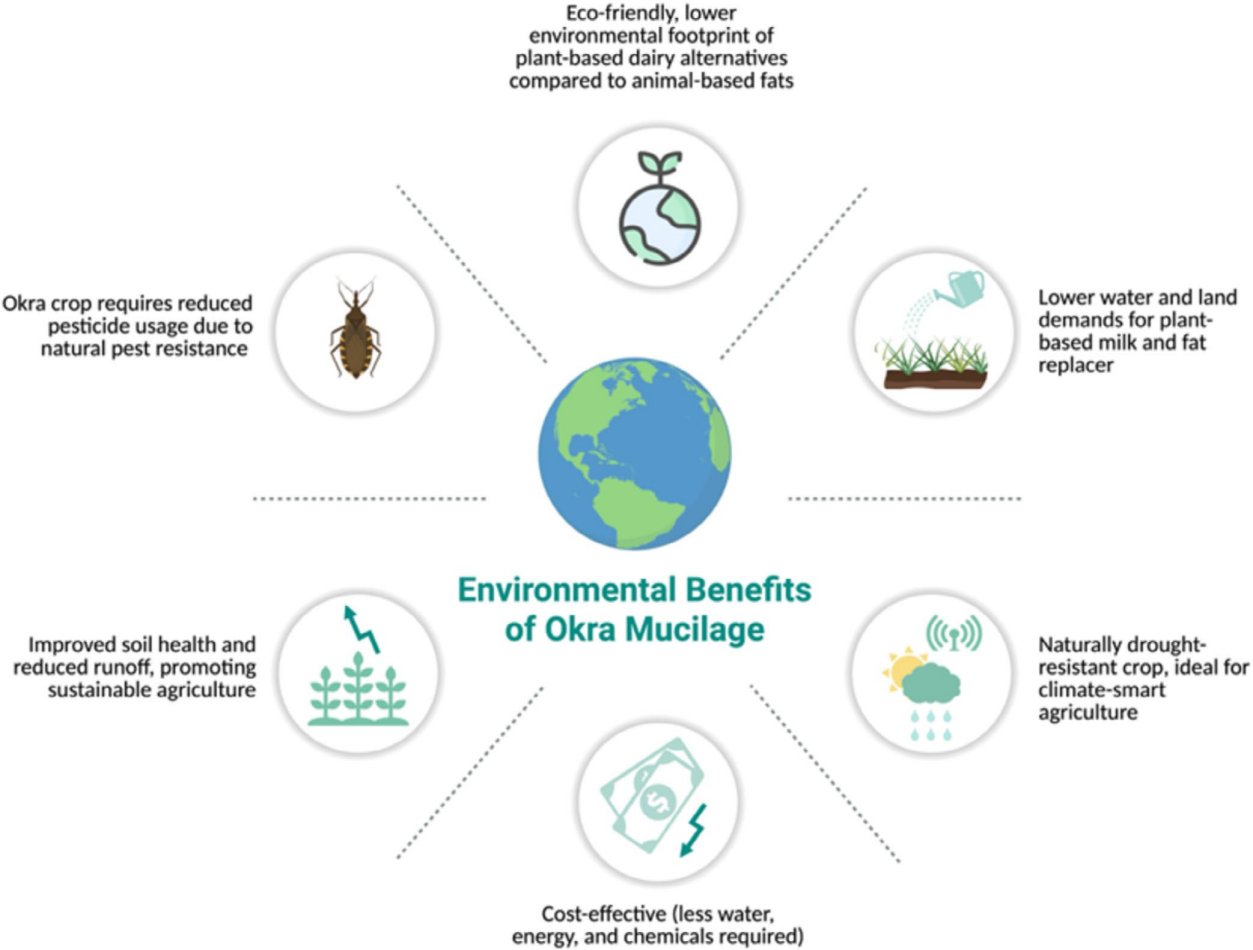
Environmental benefits of OM.

Okra is a drought‐resistant plant that flourishes in low moisture environments. Its production requires minimum pesticide use owing to its resistance to numerous pests and diseases, thus fostering sustainable agricultural practices and diminishing environmental impact through enhanced soil quality and decreased runoff (Figure 4) (Davis 2022). These attributes further strengthen the use of OM in clean‐label, sustainable food products such as reduced‐fat mozzarella cheese. Figure 4 shows the environmental benefits of OM.

#### 5.1.1 Reduction of Dairy Fat Reliance and Contribution to Plant‐Based Solutions

There has been a global shift from dairy fat to plant‐based alternatives, driven by health and environmental issues. Plant‐based dairy alternatives (PBDAs) are gaining popularity because they have a reduced environmental footprint and saturated fat content (Craig et al. 2023). Compared to dairy milk, PBDAs have lower greenhouse gas emissions (59%–71%) and require less land and water use, although they may also vary in nutritional content (Craig et al. 2023). Overconsumption of saturated fatty acids, which is mainly done through animals such as milk, has been highly linked with elevated LDL‐C levels and cardiovascular diseases (Vasilopoulou 2018). Saturated fat consumption needs to be limited, and additional plant lipids, such as monounsaturated fatty acids, can be incorporated into the diet either by modifying dairy cow nutrition or by modifying the composition using plant‐based ingredients (Vasilopoulou 2018). Furthermore, research has indicated that milk and plant‐source proteins can help mitigate the effects of lipophilic irritants, such as capsaicin. Full‐fat milk has been shown to be more effective than fat‐free milk, which implies that both the fat and protein components of full‐fat milk play a role in its functionality (Gaiser and Hayes 2023).

Legume‐based, nut‐based, cereal‐based, seed‐based, and pseudocereal‐based milk substitutes are widely recognized alternatives to dairy milk. They are healthier due to the fact that they are rich in bioactive compounds, even though their nutritional composition, sensory characteristics, and stability are significantly different from those of dairy milk (Romulo 2022). Vegetarian technologies are still being developed to replicate the nutritional and functional properties of animal‐derived foods. While total substitution with vegetarian diets leads to reduced intake of omega‐3 fatty acids and changes in mineral composition, partial or full integration has been reported to reduce saturated fats and increase dietary fiber. Some products also affect the sodium and calcium balance depending on the plant source used (Marchese et al. 2024). To this extent, OM is the top contender among vegetable fat replacers. It can mimic fat‐like texture and moisture‐holding properties, which are promising in dairy reformulation, such as LFMC, and enable sustainable and clean‐label product innovation.

#### 5.1.2 Utilization of Okra Residue Post‐Mucilage Extraction

Following the removal of mucilage from okra pods, an important quantity of residual biomass is left behind, primarily in the fibrous pod material, peels, and seeds. In most cases, such a by‐product is either disposed of or reused as animal feed. Although these practices carry some value for utility, they are sustainability problems, particularly where okra is processed on a large scale for industrial food use, such as fat replacement in LFMC. The disposal of residue squanders the environmental benefits of OM utilization and contributes to biomass waste. Several recent valorization alternatives for okra residue are aligned with circular bioeconomy and zero‐waste principles. Residual fiber can be employed as an effective source of dietary fiber in food products, providing support for gut health and texture enhancement (Feng et al. 2021). Fibrous materials have also been reported to be beneficial in biocomposite production, such as biodegradable packaging materials and films, owing to their lignocellulosic content and polymer binding capacity (Gurupranes et al. 2023). Second, okra residues are also applicable as substrates for enzyme or bioethanol production, connecting the renewable energy and bioprocessing sectors (Feng et al. 2021). The implementation of these strategies together in the OM supply chain would significantly enhance the environmental sustainability and economic value of okra‐based fat replacers. A holistic approach involving both mucilage utilization and residue valorization achieves a maximum waste generation of zero and contributes to the overall goals of sustainable food production.

### 5.2 Cost‐Effectiveness Compared to Traditional Fat Replacers

Plant‐based fat replacers are becoming increasingly important because they are often cheaper than traditional fat substitutes. Furthermore, these plant‐based foods do not compromise quality and have a lower environmental footprint than their animal‐derived counterparts do. Thus, these foods are beneficial to human health and environmental sustainability.

The production of plant‐based fat replacers may yield long‐term cost savings owing to reduced environmental compliance expenses and potential health benefits, which could lower healthcare costs linked to diet‐related diseases (Zhang, Zhang, et al. 2024).

The incorporation of plant‐based ingredients can reduce production costs and improve market competitiveness. Innovative food production methods demonstrate enhanced profitability for plant‐based products (Agibalov et al. 2022).

The manufacture of plant‐based milk can be more economical for resource utilization, including reduced water and land demand (Carlsson Kanyama et al. 2021). The initial expenditure on technology and fortification procedures may increase expenses, rendering them less accessible to some consumers (Suryamiharja et al. 2024).

### 5.3 Industrial Scalability and Challenges

OM, along with other extraction methods, is very suitable for its extensive extraction and application in dairy processing. As a natural polysaccharide, OM possesses important characteristics, such as high viscosity and heat resistance, which make it applicable for the process improvement of dairy products such as yoghurt. Ultrasound‐assisted extraction techniques provide large quantities of mucilage with beneficial thermal properties that are necessary for industrial use (Fatima et al. 2024).

The use of OM for microencapsulation of *Lactobacillus plantarum* in probiotic yoghurt illustrates its usefulness in dairy production, which is partly a function of its physicochemical and sensory properties. However, more work is needed to determine its economic practicality. There is a great possibility of using OM for large‐scale extraction and its application in dairy processing. The applicability of these options is crucial to the industry in terms of the emulsifying and stabilizing properties of plant‐based matter, which may improve product quality and shelf life (Pourakbar et al. 2023).

While the industrial production of OM has many potential benefits, it faces challenges that restrict its implementation. These include inefficiencies during extraction, lack of uniformity in quality, and limitations in the automation of production processes. There are several problems in scaling up the extraction process from the lab to industrialization, including the need for specific equipment and the handling of large volumes of raw material. Concerns about the cost‐effectiveness of supply‐sensitive regions come from the fact that the costs involved in extracting and processing the material may not be favorable in comparison to using synthetic options (Ani et al. 2012). The extraction process is such that increasing the amount of energy used achieves greater results and increases the possibility of being economical, which is suitable for a more industrialized economy (Öncü Glaue et al. 2023).

Mucilage extracted from okra, particularly at maturity index 2, exhibits considerable chemical emulsification as well as stability, meaning it can be extracted in bulk and used as an organic emulsifying agent in the dairy industry, promoting the stability of food and improving its quality (Noorlaila et al. 2015). The functional attributes and prebiotic potential of okra make it suitable for large‐scale extraction and application in dairy processing, particularly for improving sensory properties and as a fat replacement (Gajadhar 2023). Owing to its high degree of solubility (83.05%) and favorable flow properties, 9.23 Pa s viscosity, OM has excellent prospects for economic extraction and use in dairy products, which would greatly benefit food manufacturing businesses (Tsopzong et al. 2024).

## 6 Challenges and Limitations in the Application of OM

The application of OM also creates problems that need to be solved before it can be successfully used in many industries. Although it is a healthy food component and food‐grade emulsifier with significant potential, there are some factors such as sensory characteristics, yield on extraction, and homogeneity that restrict its extensive acceptance. They need to be resolved to make OM more viable and acceptable to consumers in food and non‐food products. OM's status as a regulator varies by country and application. In most regions, vegetable‐grade plant mucilage is subject to general food safety guidelines set by organizations such as the U.S. FDA, EFSA in the European Union, and Codex Alimentarius standards. Although okra is widely consumed as a vegetable, its mucilage applied in processed milk foods may require additional evaluation for labeling, functional claims, and levels of use (Richardson et al. 2013). Clearance by the responsible regulatory bodies for use as a fat substitute would depend on the established safety and efficacy through compositional data and toxicity studies. Thus, product design involving OM would be in accordance with food laws applicable to the foods of interest.

### 6.1 Sensory and Consumer Perception

OM has numerous drawbacks with regard to sensory and consumer perception applications. OM is characterized by its thickening properties and its ability to inhibit clay swelling (Murtaza et al. 2022). Seldom do people refer to an Okra containing dish as non “slimy.” Stringy and viscous are two terms that define the complex sensorial properties and are best used to describe this case. Instrumental measurement alone is insufficient for forecasting consumer appeal because of the multifaceted nature of the problem (Savouré et al. 2021). To analyze the culinary potential of OM, a multifaceted approach combining trained sensory evaluators and consumer panels is required (Francis and Williamson 2015). Its distinctive texture stems from the high concentration of polysaccharides, especially rhamnogalacturonan, which is responsible for its thick viscous texture. This remarkable texture influences the overall relief of the shape element of the dish for people suffering from dysphagia or xerostomia (Yuan et al. 2018b).

#### 6.1.1 Consumer Acceptance of OM in Traditional Dairy

OM is becoming more widely accepted in traditional dairy products owing to its useful properties and pleasant qualities. Research has shown that adding OM can enhance the quality and taste of various dairy items, such as yoghurt and frozen desserts. This growing recognition is helping promote their use in these products.

Incorporating 0.2% okra pectin into yoghurt has been shown to enhance consumer preference, although this concentration has not yet been confirmed to provide significant health benefits (Tobil et al. 2020). These findings underscore the possibility of OM improving the quality of traditional dairy products. In another study, Zayan incorporated 0.05% OM into Kareish cheese and argued that it improved the cheese's texture and sensory attributes, adding to consumer acceptance, which was linked to better organoleptic properties and nutritional advantages, especially fiber, and improved the ethnocultural attributes of the product (Zayan 2016).

Okra gum has been successfully used to completely replace milk fat in chocolate frozen dairy desserts without compromising overall acceptability or improving stability (Romanchik‐Cerpovicz et al. 2006). Nazni and Vigneshwar (2014) emphasized that OM boosts the nutritional value of dairy products because it contains high levels of proteins and soluble solids.

### 6.2 Technological Limitations

Cheese production presents many problems related to the efficiency and quality of output. This arises from a variety of issues such as the quality of the raw materials, production techniques employed, and equipment design. Understanding these limitations is important for improving cheese‐making techniques and the final product.

Technological parameters, including melting temperature, holding time, agitation speed, homogenization, cooling rate, and storage conditions, are essential for overcoming processing challenges and limitations in cheese production, profoundly influencing the structure and properties of processed cheeses (Černíková et al. 2022).

The incorporation of OM modifies the textural characteristics of cheese, thereby influencing its hardness, chewiness, and cohesion. Optimal concentrations (0.05%) enhance texture; however, elevated concentrations may result in adverse alterations. OM can improve moisture retention; however, excessive quantities may result in a soggy texture, adversely affecting the overall quality of cheese (Zayan 2016).

Technological constraints in cheese production include difficulties in formulating and validating calibration models, instrument variability, and maintaining sanitary design and compatibility with processing environments, which impede extensive implementation of process analytical technology (PAT) in the dairy sector (Panikuttira et al. 2018).

The efficacy of OM in preserving probiotic viability during storage varies. Although it may improve survival rates, the specific conditions of cheese production do not consistently promote optimal probiotic levels (Pourakbar et al. 2023).

## 7 Future Directions and Opportunities for Research

Cheese resembles traditional mozzarella as much as possible to enhance its texture and flavor. This may require changing the amount of OM used, tweaking other recipes, or trying something different altogether. Furthermore, in the case of low‐fat mozzarella cheese, it is possible to increase the vitamin and mineral contents to enhance the product.

Advanced techniques such as microencapsulation can help protect OM and enable its controlled release, improve its uniform distribution within the cheese matrix, and potentially enhance its effectiveness as a fat replacer. In addition, long‐term storage studies are essential to evaluate the impact of OM on the shelf life and quality of mozzarella cheese over an extended period of time. Furthermore, we conduct comprehensive market research analyzing consumer preferences. Sensory evaluation tests provide constructive information that will aid in restructuring the product, especially the packaging design. Conduct clinical research to assess the physiological effects of eating mozzarella cheese supplemented with OM.

Focus on important areas, such as cholesterol levels, heart health, and weight management. These studies can help to determine how this combination may positively impact overall health.

## 8 Conclusion

The food processing sector aims to produce value‐added wholesome products with lower calorie content and lower health risks. Although dairy foods, particularly cheese, are nutritious, their high fat content has deterred certain consumers from eating them owing to the prospect of health issues. However, reducing fat in mozzarella cheese would sacrifice its texture and functional properties. The incorporation of OM as a carbohydrate‐based fat substitute in LFMC offers a good solution for these issues. Studies have shown that OM, even at low levels, may improve certain textural and sensory attributes of LFMC and assist in fat reduction. This method may decrease health problems related to high fat intake without sacrificing essential traits, such as meltability and stretchability. Nevertheless, the effects in these studies with minimal usage levels are limited, and texture uniformity and consumer acceptance remain problematic. Further research is required to optimize formulations and processing conditions to exploit the potential of OM in cheese products with respect to quality and consumer acceptability.

## Author Contributions

**Rabbia Khan:** writing – original draft (equal). **Ali Ikram:** supervision (equal). **Muhammad Tayyab Arshad:** writing – review and editing (equal). **Feroza Naveed:** data curation (equal). **Md. Sakhawot Hossain:** data curation (equal), writing – review and editing (equal). **Sammra Maqsood:** formal analysis (equal). **Hatem A. Al‐Aoh:** conceptualization (equal), visualization (equal). **Kodjo Théodore Gnedeka:** validation (equal).

## Ethics Statement

The authors have nothing to report.

## Consent

The authors have nothing to report.

## Conflicts of Interest

The authors declare no conflicts of interest.

## Data Availability

The data that support the findings of this study are available from the corresponding author upon reasonable request.
